# Supplementary figures and images for: Genetic code expansion, click chemistry, and light-activated PI3K reveal details of membrane protein trafficking downstream of receptor tyrosine kinases
Source: eLife. 2024 Aug 20;12:RP91012. doi: 10.7554/eLife.91012 (PMC11335347; doi:10.7554/eLife.91012)

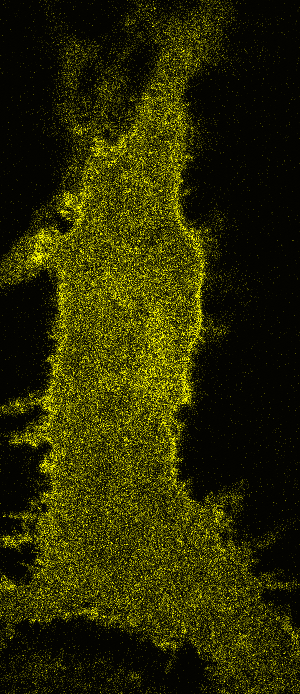

Supplement: Figure 1—figure supplement 1—source data 1. [file elife-91012-fig1-figsupp1-data1.zip › 91012Figure1S1SourceData1/Figure 1-figure supplement 1-source data 1 Frame2.tif]

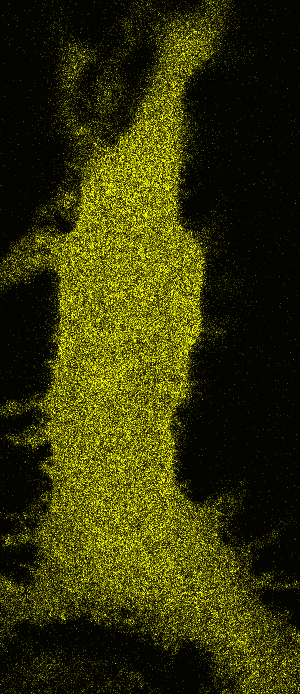

Supplement: Figure 1—figure supplement 1—source data 1. [file elife-91012-fig1-figsupp1-data1.zip › 91012Figure1S1SourceData1/Figure 1-figure supplement 1-source data 1 Frame3.tif]

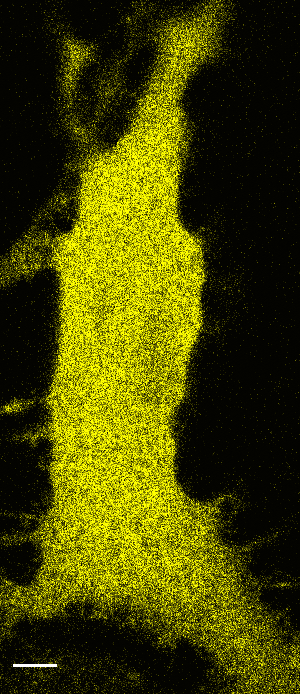

Supplement: Figure 1—figure supplement 1—source data 1. [file elife-91012-fig1-figsupp1-data1.zip › 91012Figure1S1SourceData1/Figure 1-figure supplement 1-source data 1 Frame1.tif]

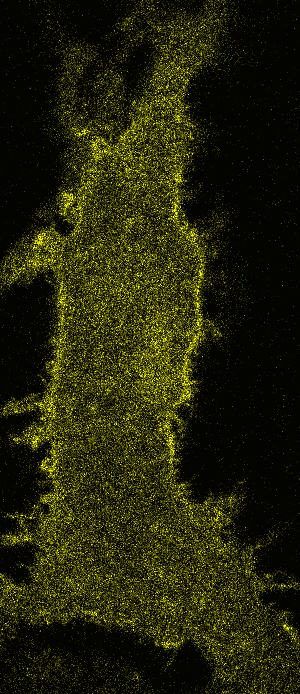

Supplement: Figure 1—figure supplement 1—source data 1. [file elife-91012-fig1-figsupp1-data1.zip › 91012Figure1S1SourceData1/Figure 1-figure supplement 1-source data 1 Frame4.tif]

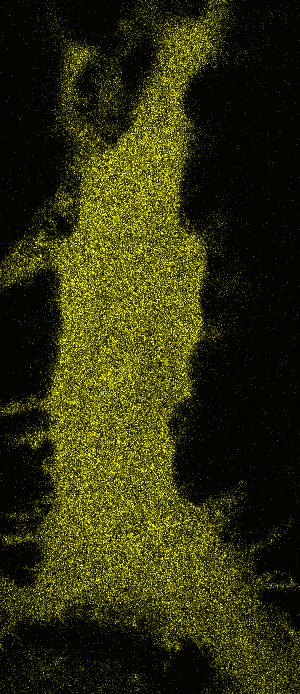

Supplement: Figure 1—figure supplement 1—source data 1. [file elife-91012-fig1-figsupp1-data1.zip › 91012Figure1S1SourceData1/Figure 1-figure supplement 1-source data 1 Frame5.tif]

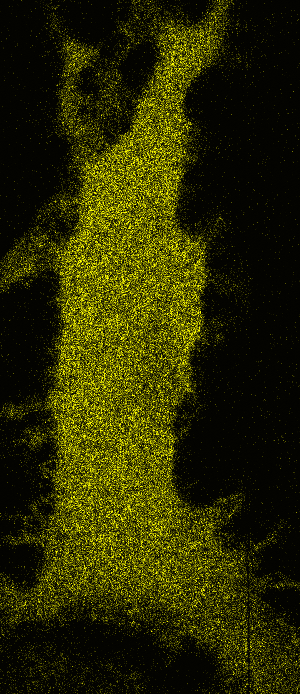

Supplement: Figure 1—figure supplement 1—source data 1. [file elife-91012-fig1-figsupp1-data1.zip › 91012Figure1S1SourceData1/Figure 1-figure supplement 1-source data 1 Frame7.tif]

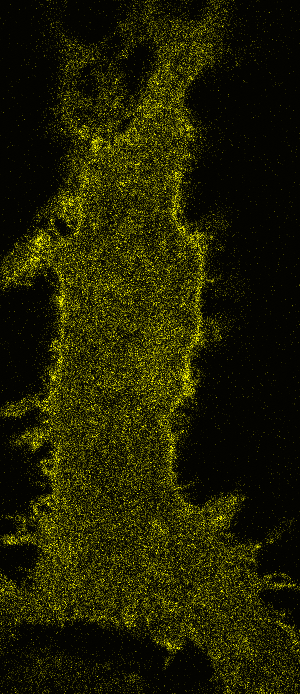

Supplement: Figure 1—figure supplement 1—source data 1. [file elife-91012-fig1-figsupp1-data1.zip › 91012Figure1S1SourceData1/Figure 1-figure supplement 1-source data 1 Frame6.tif]

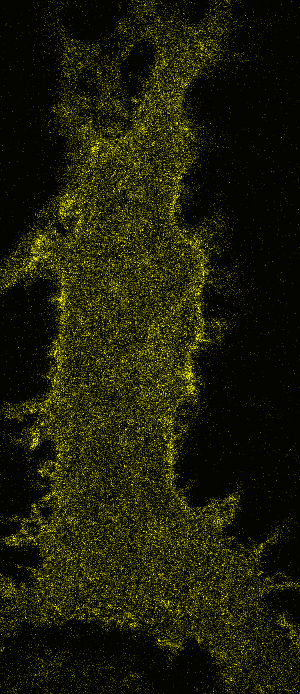

Supplement: Figure 1—figure supplement 1—source data 1. [file elife-91012-fig1-figsupp1-data1.zip › 91012Figure1S1SourceData1/Figure 1-figure supplement 1-source data 1 Frame8.tif]

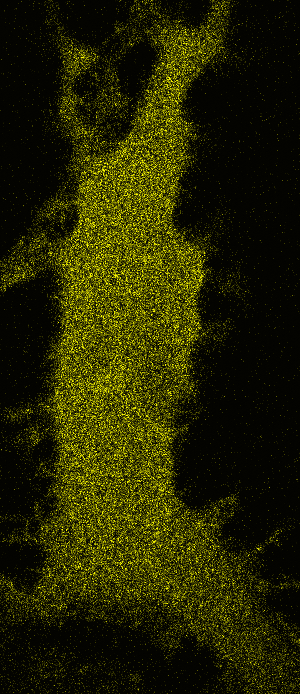

Supplement: Figure 1—figure supplement 1—source data 1. [file elife-91012-fig1-figsupp1-data1.zip › 91012Figure1S1SourceData1/Figure 1-figure supplement 1-source data 1 Frame9.tif]

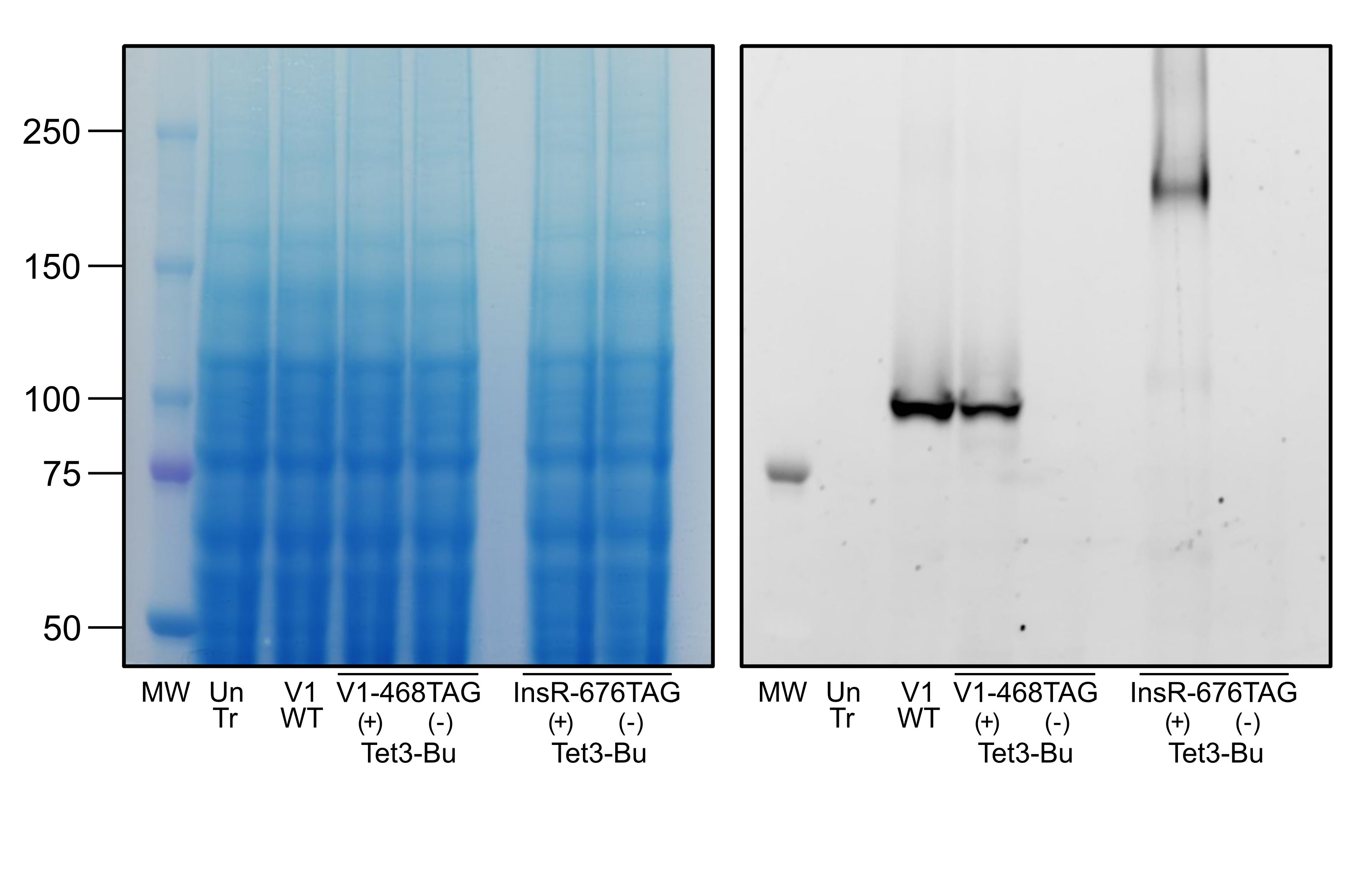

Supplement: Figure 3—figure supplement 1—source data 3. [file elife-91012-fig3-figsupp1-data3.zip › Figure 3-figure supplement 1-source data 1 Commassie_GFP_labeled.tif]

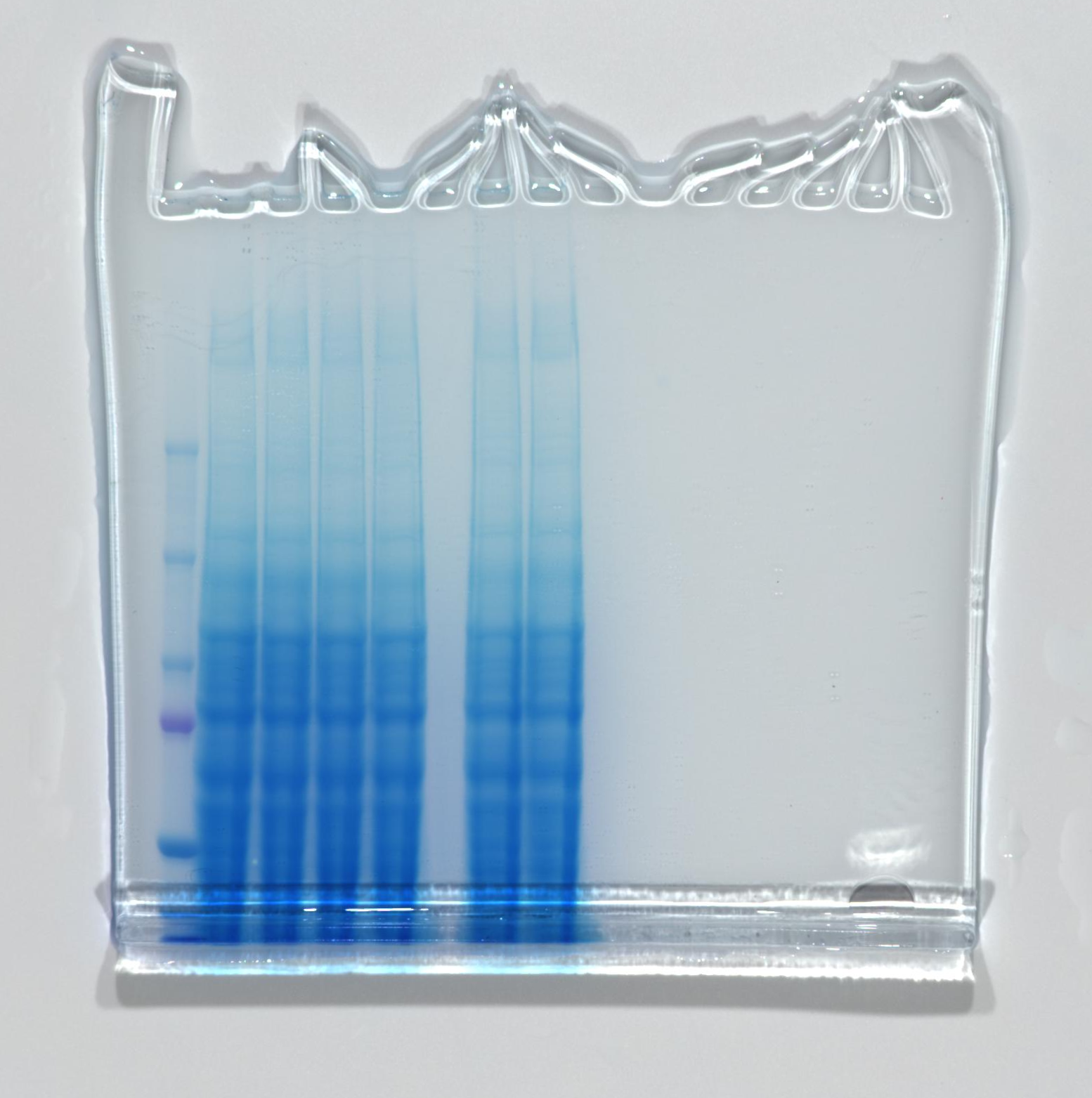

Supplement: Figure 3—figure supplement 1—source data 3. [file elife-91012-fig3-figsupp1-data3.zip › Figure 3-figure supplement 1-source data 1 Coomassie_Original Gel.tif]

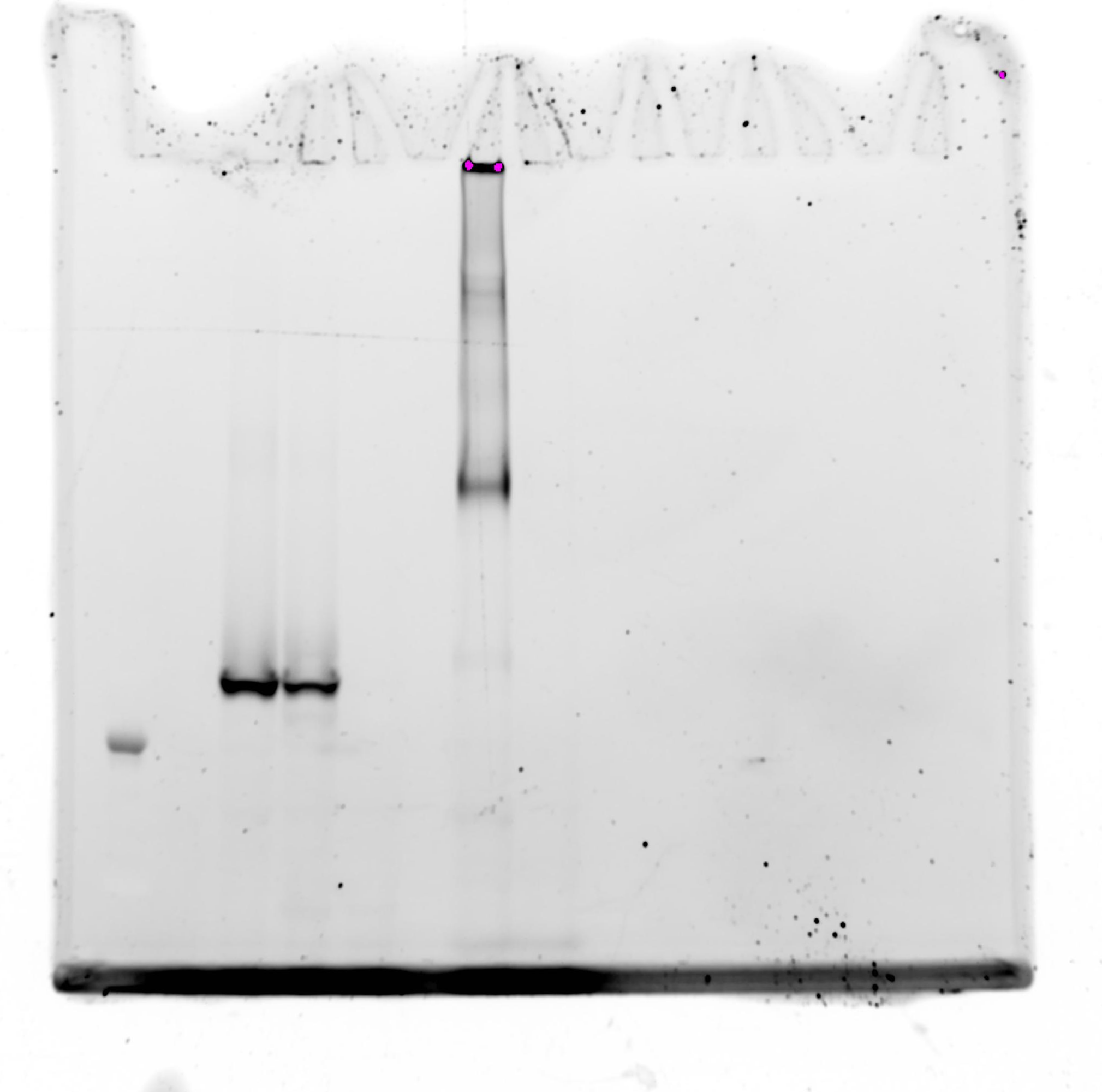

Supplement: Figure 3—figure supplement 1—source data 3. [file elife-91012-fig3-figsupp1-data3.zip › Figure 3-figure supplement 1-source data 1 GFP_Original Gel.tif]
